# Genome-wide identification reveals unprecedented expansion and stress-responsive expression patterns of the lipoxygenase gene family in soybean (*Glycine max* L.)

**DOI:** 10.3389/fpls.2025.1702499

**Published:** 2025-12-03

**Authors:** Sobhi F. Lamlom, Taifeng Zhang, Yanbo Wang, Honglei Ren, Fuqiang Song, Guojun Feng

**Affiliations:** 1 Work Station of Science and Technique for Post-doctoral in Sugar Beet Institute, Heilongjiang University, Harbin, China; 2 Heilongjiang Academy of Agricultural Sciences, Soybean Research Institute, Harbin, China; 3 College of Advanced Agriculture and Ecological Environment, Heilongjiang University, Harbin, China; 4 Postdoctoral Research Station in Ecology, School of Life Sciences, Heilongjiang University, Harbin, China; 5 Plant Production Department, Faculty of Agriculture Saba Basha, Alexandria University, Alexandria, Egypt; 6 Heilongjiang Junyi Agricultural Limited Liability Company, Harbin, China

**Keywords:** soybean, lipoxygenase, abiotic stress, gene expression, phylogenetic analysis, synteny, oxylipin biosynthesis, stress tolerance

## Abstract

**Background:**

Lipoxygenases (LOXs) catalyze polyunsaturated fatty acid oxygenation and regulate plant stress responses and oxylipin biosynthesis. This study provides comprehensive genome-wide characterization of the soybean LOX gene family, revealing unprecedented expansion and functional diversification.

**Methods:**

We identified unique *GmLOX* gene loci through systematic database searches and domain analysis, selecting representative isoforms to avoid transcript redundancy. Phylogenetic analysis compared 43 *GmLOX* with 6 *AtLOX* proteins. Protein structures, conserved domains, promoter cis-regulatory elements (2,000 bp upstream), and synteny relationships were characterized. Expression profiling under four abiotic stresses (alkaline, drought, heat, salt) across six time points (0–24 h) was performed using qRT-PCR.

**Results:**

Genes distribute unevenly across 10 chromosomes, with highest densities on Gm10 (11 genes), Gm08 (9 genes), and Gm07 (8 genes). Phylogenetic analysis revealed six evolutionary clades with soybean-specific expansion. Structural analysis showed 92% encode full-length proteins (average 887 amino acids) with conserved PLAT/LH2 (97%) and catalytic domains. Promoter analysis identified 23 regulatory element categories, including hormone-responsive (auxin 97%, gibberellin 89%, ABA 94%), stress-responsive (95%), and light-responsive elements (100%). Synteny analysis demonstrated ancient whole-genome duplications and recent tandem duplications drove expansion. Expression profiling revealed five functional categories: alkaline-specific, drought-dominant, heat-specific, salt-responsive, and multi-stress responsive genes. *GmLOX16* showed exceptional multi-stress responsiveness (40-fold salt, 38-fold alkaline), *GmLOX13* displayed strongest heat response (20-fold), and *GmLOX17* exhibited peak drought sensitivity (7-fold).

**Conclusion:**

Soybean possesses the largest crop LOX family with remarkable functional diversification maintained under strong selective pressure (92% functional retention). Key stress-responsive genes, particularly *GmLOX16* and *GmLOX13*, represent valuable targets for molecular breeding to enhance climate resilience, providing critical genetic resources for sustainable crop improvement.

## 1 Introduction

Soybean (*Glycine max* (L.) Merr.) is one of the most economically important leguminous crops worldwide, providing essential protein and oil for human consumption and animal feed. Global soybean production exceeded 370 million metric tons in 2023, accounting for approximately 60% of the world’s oilseed production and serving as a critical component of food security systems (FAO, 2024). However, as climate change intensifies, soybean agriculture faces mounting challenges from abiotic stresses including drought, salinity, extreme temperatures, and heavy metal toxicity (Zhu et al., 2024). These environmental limitations have a significant effect on the productivity and quality of soybeans, which puts global food security and the long-term health of farming at risk (Feng et al., 2020; Zhu et al., 2024). Understanding the molecular mechanisms governing soybean responses to abiotic stress is therefore essential for developing climate-resilient cultivars capable of maintaining productivity under increasingly variable environmental conditions (Sarkar et al., 2023).

Lipoxygenases (LOXs; EC 1.13.11.12) are non-heme iron-containing dioxygenases that catalyze the stereospecific oxygenation of polyunsaturated fatty acids (PUFAs) containing a (1Z,4Z)-pentadiene system (Chedea and Jisaka, 2011; Vick, 2018). These enzymes serve as critical regulators in the oxylipin biosynthesis pathway, generating fatty acid hydroperoxides that are subsequently converted into bioactive signaling molecules including jasmonic acid (JA), green leaf volatiles (GLVs), and other oxylipins (Shafi et al., 2023). LOX-derived oxylipins play indispensable roles in plant stress responses, developmental regulation, and defense signaling networks. Plant LOX proteins share two conserved structural domains: an N-terminal PLAT/LH2 (Polycystin-1, Lipoxygenase, Alpha-Toxin) domain involved in membrane binding and substrate positioning, and a C-terminal catalytic domain containing the iron-binding histidine-rich motif essential for enzymatic activity (Porta and Rocha-Sosa, 2002; Malviya et al., 2023).

Based on their positional specificity of fatty acid oxygenation, plant LOXs are classified into two major groups: 9-LOX and 13-LOX (Porta and Rocha-Sosa, 2002; Malviya et al., 2023). The 9-LOX enzymes oxygenate linolenic acid at the C-9 position, producing 9-hydroperoxy-(10E,12Z)-octadecatrienoic acid (9-HPOT), which contributes to lateral root development and specific stress responses (Porta and Rocha-Sosa, 2002). In contrast, the 13-LOX pathway is crucial for plant stress responses through the production of 13-hydroperoxy-(9Z,12E)-octadecatrienoic acid (13-HPOT), the precursor for JA biosynthesis (Wang et al., 2008; López et al., 2011). Jasmonic acid and its derivatives function as master regulators of plant defense responses, wound signaling, and adaptation to environmental stresses, making the 13-LOX pathway particularly important for stress tolerance mechanisms (Kolomiets et al., 2001; Li et al., 2025).

Recent genome-wide studies have revealed extensive variation in LOX gene family size and functional specialization across plant species. *Arabidopsis thaliana* possesses six LOX genes (Umate, 2011), rice (*Oryza sativa*) contains 14 LOX genes (Wang et al., 2008), maize (*Zea mays*) has 13 members (Li et al., 2025), poplar harbors 21 LOX genes (Chen et al., 2015), and chickpea (*Cicer arietinum*) contains 15 LOX genes (Malviya et al., 2023). This considerable variation in gene family size suggests that LOX gene expansion has occurred independently in different lineages, potentially reflecting lineage-specific adaptations to environmental pressures. However, despite soybean’s economic importance and complex genome architecture resulting from multiple polyploidization events, a comprehensive characterization of the soybean LOX gene family and its specialized functions in abiotic stress responses remains lacking.

In legumes, LOX genes may perform additional specialized functions related to nodulation and nitrogen fixation—processes that are particularly sensitive to abiotic stress conditions (Porta and Rocha-Sosa, 2002; Veronico et al., 2006). The symbiotic relationship between soybean and rhizobia bacteria is severely compromised under stress conditions, potentially involving LOX-mediated signaling pathways that regulate nodule development and function (Singh et al., 2022). Furthermore, oxylipins produced through LOX activity have been implicated in regulating the establishment and maintenance of legume-rhizobia symbiosis, suggesting that the expanded LOX gene repertoire in soybean may reflect evolutionary adaptations specific to legume biology.

The complex paleopolyploid genome of soybean, resulting from two ancient whole-genome duplication (WGD) events approximately 59 and 13 million years ago, has provided extensive opportunities for gene family expansion and functional diversification (Nadon, 2019). Following WGD events, duplicated genes may undergo several evolutionary fates: pseudogenization (loss of function), subfunctionalization (partitioning of ancestral functions), or neofunctionalization (acquisition of novel functions) (Panchy et al., 2016). The retention and diversification of duplicated LOX genes in soybean may have enhanced the species’ adaptive capacity to environmental challenges by enabling more sophisticated regulation of oxylipin-mediated stress responses. Understanding how soybean LOX genes have evolved and diversified following these duplication events can provide insights into the mechanisms underlying gene family evolution in polyploid crop genomes. Understanding the stress-responsive functions of soybean LOX genes represents a practical approach to crop improvement. By elucidating the molecular pathways enabling LOX-mediated stress tolerance, researchers can develop targeted breeding strategies or biotechnological interventions to enhance soybean resilience. Recent advances in genome editing technologies, such as CRISPR/Cas9-mediated modification of specific LOX genes for improved seed quality (Patel et al., 2025), demonstrate the potential for precision engineering of LOX functions. However, such targeted approaches require comprehensive functional characterization of individual family members to identify optimal targets for manipulation without compromising essential physiological functions.

In this study, we conducted a comprehensive genome-wide identification and characterization of LOX genes in soybean, examining their evolutionary relationships, structural features, chromosomal distribution, regulatory elements, and expression patterns under multiple abiotic stress conditions. Our objectives were to: (1) identify and characterize all LOX gene family members in the soybean genome; (2) investigate their evolutionary origins through phylogenetic and synteny analyses; (3) analyze conserved protein domains, gene structures, and promoter regulatory elements; and (4) determine their expression responses to alkaline, drought, heat, and salt stress conditions. Our findings provide valuable insights into the evolutionary diversification and functional specialization of soybean LOX genes, establishing a foundation for developing molecular strategies to enhance soybean tolerance to abiotic stress in the context of global climate change.

## 2 Materials and methods

### 2.1 Genome-wide identification of *GmLOX* gene family members

Soybean LOX gene family members were identified through comprehensive database searches using BLAST algorithms on NCBI (http://www.ncbi.nlm.nih.gov) and Phytozome v13 (https://phytozome.jgi.doe.gov/pz/portal.html). Candidate genes were screened for conserved domains characteristic of Lipoxygenases (LOXs; EC 1.13.11.12) using SMART domain analysis (http://smart.embl-heidelberg.de). The candidate genes were screened for the presence of conserved domains characteristic of lipoxygenases using the SMART database. Genes containing the definitive Lipoxygenase (PF00305) and PLAT (PF01477) domains and showing high amino acid sequence similarity to known *Arabidopsis thaliana* LOX proteins were selected as bona fide soybean LOX family members. The physicochemical properties of the identified LOX proteins, including molecular weight, isoelectric point, and instability index, were analyzed using ExPASy ProtParam (https://web.expasy.org/protparam/). Subcellular localization predictions were performed using Cell-PLoc 2.0 (http://www.csbio.sjtu.edu.cn/bioinf/Cell-PLoc-2/).

### 2.2 Phylogenetic analysis and protein domain architecture

LOX protein sequences from *A. thaliana* and *Oryza sativa* were obtained from Phytozome based on EC classification (1.2.1.44) and conserved domain analysis. According to comprehensive annotation in TAIR10 and phylogenetic studies (Umate, 2011), *Arabidopsis thaliana* contains six LOX genes: AtLOX1 (AT1G55020), AtLOX2 (AT3G45140), AtLOX3 (AT1G17420), AtLOX4 (AT1G72520), AtLOX5 (AT3G22400), and AtLOX6 (AT1G67560). All six genes were included in our phylogenetic analysis. We verified this completeness through BLAST searches against the TAIR database using conserved LOX domains (PF01370) with E-value < 1e-10, confirming no additional LOX genes exist in the *Arabidopsis* genome. Multiple sequence alignments were performed using MUSCLE, and phylogenetic relationships were inferred with the neighbor-joining method implemented in MEGA11 with 1,000 bootstrap replicates. Phylogenetic trees were visualized and annotated using iTOL (http://itol.embl.de). Protein domain architecture was analyzed using Phytozome annotations and visualized with IBS software to display domain organization and conservation patterns across family members.

### 2.3 Motif composition and gene structure analysis

Conserved motifs in soybean LOX proteins were identified using the MEME Suite (https://meme-suite.org/meme/tools/meme) with default parameters, limited to 10 motifs. Gene structure analysis, including exon-intron organization, was performed using genome annotation files downloaded from Phytozome v13. Both motif distribution and gene structure were visualized with TBtools software.

### 2.4 Promoter analysis and cis-regulatory elements

Promoter sequences (2,000 bp upstream of the translation start site) for each GmLOX gene were retrieved from the Phytozome database. Cis-acting regulatory elements were predicted using PlantCARE (https://bioinformatics.psb.ugent.be/webtools/plantcare/html/), focusing on stress-responsive, hormone-responsive, and tissue-specific elements. Results were visualized with TBtools for comparative analysis among family members.

### 2.5 Synteny and collinearity analysis

The syntenic relationships of soybean LOX genes were examined both within the soybean genome (via segmental duplications) and between soybean and other plant species. Collinearity analysis was conducted using TBtools with default settings to identify orthologous and paralogous gene pairs and to visualize syntenic blocks. LOX protein sequences from *Zea mays* were retrieved from Phytozome v13 (www.phytozome.net) using the same EC classification (1.13.11.12) and conserved domain criteria applied to *A. thaliana* and *O. sativa*, and were included in the multi-species synteny analysis.

### 2.6 Plant material, growth conditions and abiotic stress treatments

Soybean cultivar Heinong 551 seeds were acquired from the Heilongjiang Academy of Agricultural Sciences, China. Seeds were surface sterilized with 1% sodium hypochlorite solution for 10 minutes, then thoroughly washed with sterile distilled water. The sterilized seeds were germinated on moist filter paper in darkness at 25 °C for 3 days. Uniform seedlings were selected and transferred to hydroponic systems containing modified Hoagland’s nutrient solution (pH 6.0) under controlled environmental conditions. Plants were grown in a growth chamber maintained at 25 ± 2 °C with a 16/8 hour light/dark cycle, 200 μmol m^−2^ s^−1^ photosynthetic photon flux density, and 60-70% relative humidity. The nutrient solution was refreshed every 3 days to ensure optimal growth conditions.

Two-week-old soybean seedlings with fully expanded trifoliate leaves were subjected to four different abiotic stress treatments. For alkaline stress, seedlings were transferred to a nutrient solution adjusted to pH 9.0 with NaOH. Drought stress was imposed by moving seedlings to a nutrient solution containing 20% polyethylene glycol (PEG-6000) to simulate osmotic stress equivalent to -0.5 MPa water potential. Heat stress involved transferring plants to a growth chamber set at 40 ± 2 °C while maintaining other environmental conditions constant. Salt stress was applied by transferring seedlings to a nutrient solution supplemented with 200 mM NaCl. Control plants were kept in a standard nutrient solution under normal growth conditions.

### 2.7 Sample Collection and RNA Extraction

Leaf samples were collected from the first trifoliate leaves at 0, 1, 3, 6, 12, and 24 hours after treatment began. Three independent biological replicates were gathered for each treatment and time point, with each replicate consisting of leaves pooled from three individual plants. Samples were immediately frozen in liquid nitrogen and stored at -80 °C until RNA extraction. Total RNA was isolated using TRIzol reagent (Invitrogen, USA) according to the manufacturer’s protocol. RNA quality and concentration were evaluated using a NanoDrop 2000 spectrophotometer (Thermo Scientific, USA) and 1% agarose gel electrophoresis. Only RNA samples with A260/A280 ratios between 1.8 and 2.2, along with clear ribosomal RNA bands, were used for further analyses. First-strand cDNA was synthesized from 1 μg of total RNA using the PrimeScript RT Reagent Kit (Takara, Japan), following the manufacturer’s instructions. The reaction was carried out at 37 °C for 15 minutes, then heated to 85 °C for 5 seconds to inactivate the reverse transcriptase. Quantitative real-time PCR (qRT-PCR) was performed using SYBR Premix Ex Taq II (Takara, Japan) on a CFX96 Real-Time PCR Detection System (Bio-Rad, USA). Each 20 μL reaction contained 10 μL of SYBR Premix Ex Taq II, 0.8 μL of each primer (forward and reverse, 10 μM), 2 μL of cDNA template (diluted 1:10), and 6.4 μL of nuclease-free water.

### 2.8 PCR Conditions and Gene-Specific Primers

The qRT- PCR cycling conditions were as follows: initial denaturation at 95 °C for 30 seconds, followed by 40 cycles of 95 °C for 5 seconds and 60 °C for 30 seconds. A melting curve analysis was performed from 65 °C to 95 °C with 0. 0.5 °C increments to verify primer specificity and the absence of primer dimers. Gene-specific primers were designed for 42 *GmLOX* genes selected to represent: (1) all six phylogenetic clades identified in our analysis; (2) diverse chromosomal locations; and (3) predicted full-length functional proteins (excluding the seven truncated genes likely representing pseudogenes). This selection strategy ensured comprehensive coverage of the functional diversity within the *GmLOX* gene family while focusing experimental resources on genes with high likelihood of biological activity. *GmLOX* genes primers were designed using Primer 3 Plus software based on gene sequences obtained from the Phytozome database (**Supplementary Table S1**). Primer pairs were designed to amplify products of 150–250 bp with melting temperatures of 58- 62 °C. The soybean *GmActin 11* gene (*Glyma. 18 G 290800*) was used as an internal reference gene for normalization because of its stable expression under various stress conditions. Relative gene expression levels were calculated using the 2 ^ (-ΔΔCt) method, where ΔΔCt = (Ct target gene - Ct reference gene) for the treatment minus the same difference for the control. Expression values are presented as fold changes relative to the untreated control samples (0 h time point). Each qRT-PCR reaction was performed in technical triplicate, and three independent biological replicates were analyzed for each treatment and time point. Statistical significance was determined using Student’ s t- test with Bonferroni correction for multiple comparisons. Differences were considered statistically significant at P < 0. 05. Data visualization and statistical analyses were conducted with GraphPad Prism 8. 0 software. All primer pairs were validated for specificity through melting curve analysis and agarose gel electrophoresis of PCR products. PCR efficiency was assessed using serial dilutions of cDNA templates, and only primer pairs with efficiencies between 90-110% and R ²> 0. 0.99 were used for expression analysis.

### 2.9 Co-expression network analysis

To investigate the potential transcriptional regulatory mechanisms of *GmLOX* genes, co-expression network analysis was performed using the GM-CX database (http://gm-cx.org/), a comprehensive soybean gene co-expression resource constructed from large-scale RNA-seq datasets encompassing diverse developmental stages, tissue types, and stress conditions [citation needed]. The GM-CX database employs mutual rank (MR)-based methods to identify significant co-expression relationships between genes across the soybean genome. Co-expression relationships between *GmLOX* genes and transcription factors were extracted from the GM-CX database using the following criteria: (1) only interactions with log-likelihood score (LLS) values ≥ 2.0 were considered significant, representing statistically robust co-expression patterns; (2) only protein-coding genes annotated as transcription factors in the soybean genome were included as potential regulators. The LLS value, which measures the strength of co-expression based on correlation coefficients and mutual rank statistics, was used as a quantitative indicator of regulatory potential, with higher LLS values indicating stronger co-expression relationships. Transcription factor annotations were obtained from the Plant Transcription Factor Database (PlantTFDB v5.0, http://planttfdb.gao-lab.org/) and cross-referenced with the Phytozome v13 soybean genome annotation. TF family classifications were assigned based on conserved DNA-binding domain architecture. The co-expression network was visualized using Cytoscape v3.9.1 (Shannon et al., 1971), with *GmLOX* genes and transcription factors represented as nodes and co-expression relationships as edges. Edge thickness was scaled proportionally to LLS values to visually represent the strength of co-expression. Network topology parameters, including degree distribution and hub gene identification, were calculated using the NetworkAnalyzer plugin in Cytoscape. To assess the biological relevance of the identified co-expression relationships, we integrated the network data with our experimental expression profiling results. *GmLOX* genes showing significant differential expression under abiotic stress treatments (fold change ≥ 2.0, p < 0.05) were cross-referenced with their co-expressed TFs to identify potential stress-responsive regulatory modules.

## 3 Results

### 3.1 Genome-wide identification and characterization of *GmLOX* genes

A comprehensive genome-wide analysis of the soybean genome identified a total of 43 LOX genes, designated as *GmLOX1* through *GmLOX43* based on their chromosomal locations (**Supplementary Table S2**). This collection represents one of the most notable LOX gene families reported in any plant species to date. The identified *GmLOX* genes were unevenly distributed across 10 of the 20 soybean chromosomes (Gm03, Gm07, Gm08, Gm10, Gm11, Gm12, Gm13, Gm16, Gm19, and Gm20), while ten chromosomes (Gm01, Gm02, Gm04, Gm05, Gm06, Gm09, Gm14, Gm15, Gm17, and Gm18) lacked LOX genes entirely. Chromosome Gm08 contained the highest number of LOX genes with 13 members, followed by chromosome Gm07 with 10 genes, Gm13 with 7 genes, Gm11 with 6 genes, Gm20 with 3 genes, and chromosomes Gm03, Gm10, Gm12, Gm16, and Gm19 with 1, 1, 1, 1, and 1 gene, respectively. All identified genes contained conserved domains typical of lipoxygenases, including the N-terminal PLAT/LH2 domain and the C-terminal catalytic domain with the essential histidine-rich motif, which is critical for iron binding and enzymatic activity. The physicochemical properties of *GmLOX* proteins showed considerable variation across family members, indicating potential functional diversification (**Supplementary Table S2**). The predicted molecular weights ranged from 30.35 kDa (*GmLOX13*) to 109.88 kDa (*GmLOX38*), with an average molecular weight of approximately 95–100 kDa for most members. The theoretical isoelectric points (pI) ranged from 5.18 (*GmLOX7*) to 9.62 (*GmLOX9*), reflecting diverse charge characteristics that may influence their subcellular localization and protein interactions. Protein lengths varied considerably from 56 amino acids (*GmLOX13*) to 995 amino acids (*GmLOX37*), with most full-length proteins containing approximately 850–950 amino acids, suggesting high structural conservation among functional members. Several genes (including GmLOX12, *GmLOX13*, and others with fewer than 200 amino acids) encoded substantially shorter proteins, likely representing pseudogenes or gene fragments resulting from incomplete duplications or degradation events. Subcellular localization predictions using multiple computational tools indicate that *GmLOX* proteins are predicted to localize primarily to the cytoplasm and chloroplast, with this dual localization aligning with the diverse metabolic functions of lipoxygenases in plant cells and suggesting their roles in both cytoplasmic and plastidial fatty acid metabolism, including the biosynthesis of oxylipins and stress-related signaling molecules such as jasmonic acid and its derivatives.

### 3.2 Phylogenetic analysis and classification of *GmLOX* genes

**Figure 1:**
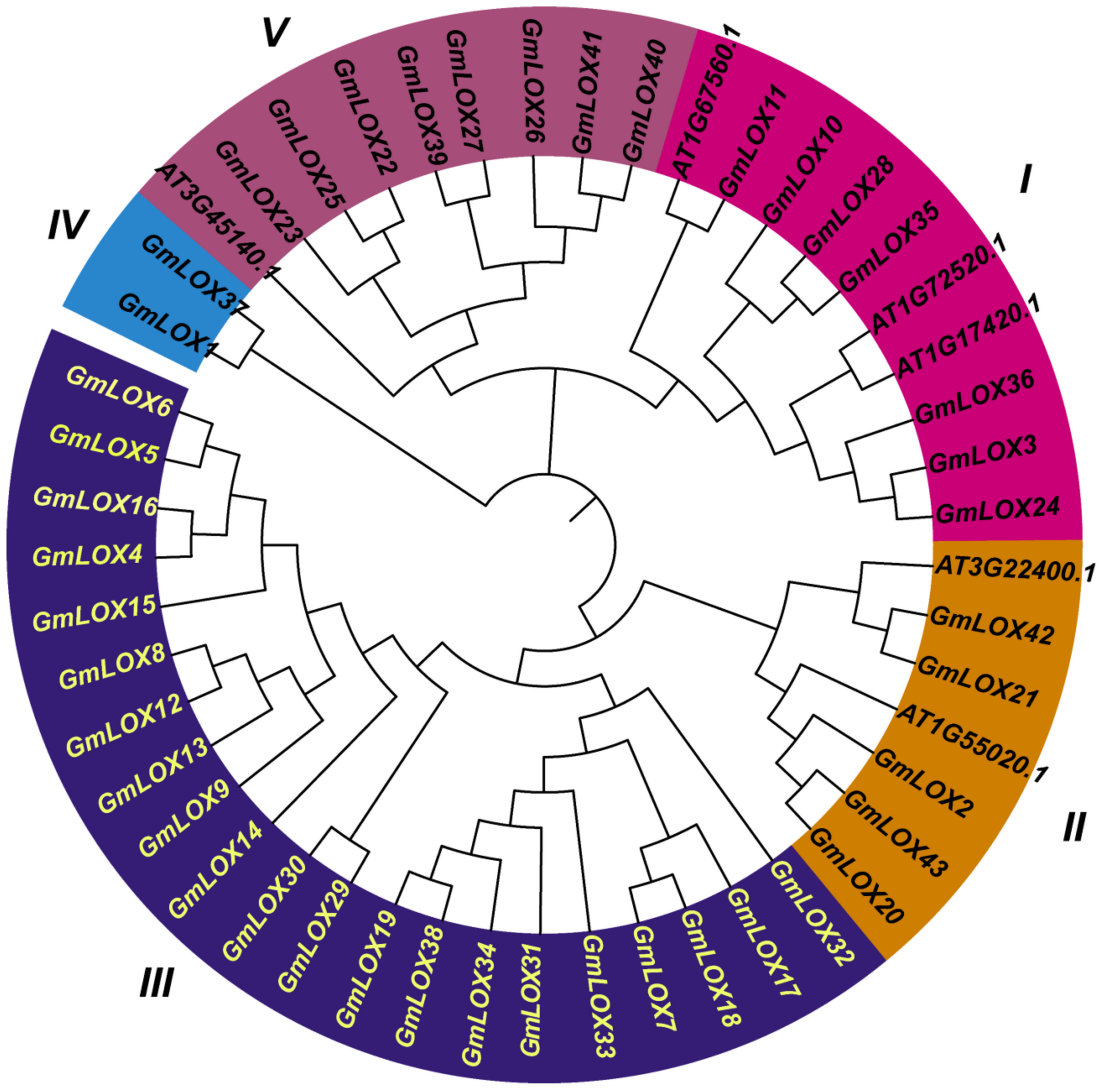
Phylogenetic relationships and evolutionary classification of lipoxygenase proteins in soybean and Arabidopsis. Neighbor-joining phylogenetic tree of 43*GmLOX* proteins from soybean (*Glycine max*) and 6 *AtLOX* proteins from *Arabidopsis thaliana* (*AT3G22400.1, AT1G55020.1, AT3G45140.1, AT1G17420.1, AT1G72520.1, AT1G67560.1*). The tree was constructed using MEGA-12 software with 1,000 bootstrap replicates, and bootstrap values >70% are shown at major nodes.

Phylogenetic analysis of the 43 *GmLOX* proteins along with six LOX sequences from Arabidopsis thaliana (*AT3G22400.1, AT1G55020.1, AT3G45140.1, AT1G17420.1, AT1G72520.1*, and *AT1G67560.1*) revealed complex evolutionary relationships. It enabled the classification of soybean LOX proteins into six distinct subfamilies (**Figure 1**). The neighbor-joining phylogenetic tree, built with 1,000 bootstrap replicates, distinctly separated LOX proteins into major evolutionary groups, with soybean LOX genes showing both orthologous relationships with Arabidopsis LOX genes and extensive soybean-specific expansions. The relationships uncovered indicated that the large expansion of the soybean LOX gene family mainly occurred through lineage-specific duplications, especially in Clade III. Bootstrap support values over 70% at key nodes confirmed the reliability of the phylogenetic results. The analysis indicates that soybean LOX genes have undergone both subfunctionalization and neofunctionalization after duplication events, as shown by the retention of multiple paralogs and their different expression patterns.

### 3.3 Conserved domain and gene structure analysis of *GmLOX* proteins

**Figure 2:**
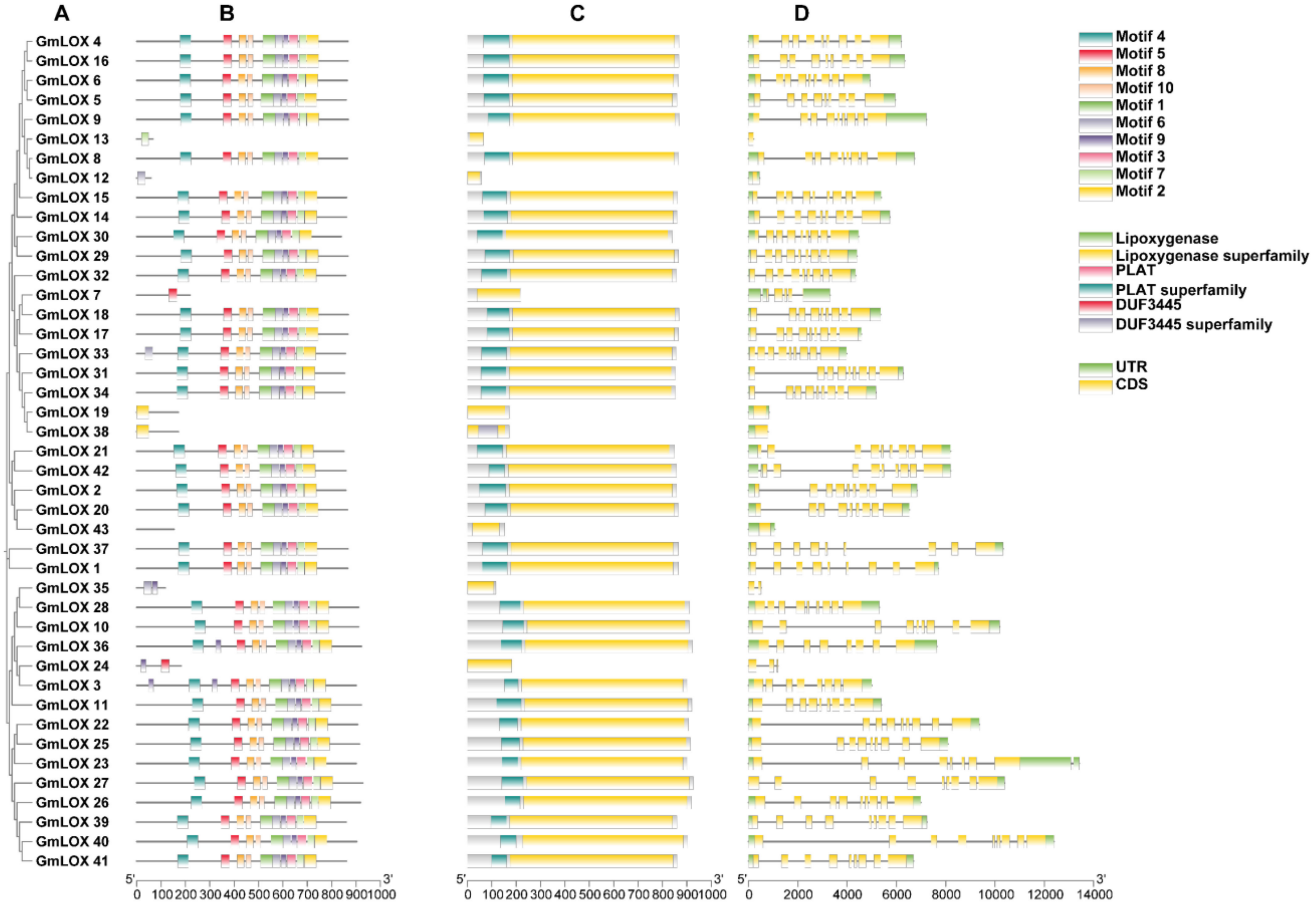
Comprehensive gene structure, domain architecture and motif organization of *GmLOX* genes and proteins. **(A)** analysis of *GmLOX* gene structure and protein organization across all 43 family members. **(B)** Gene structure represents exon-intron organization with motif distribution patterns. Each horizontal line represents a gene with colored boxes indicating the 10 conserved motifs (Motif 1-10). **(C)** Simplified gene structure view highlighting the overall organization and relative positions of structural elements across the gene family. Various colored elements show the positions of conserved domains, including lipoxygenase domain, lipoxygenase superfamily domain, PLAT domain, PLAT superfamily domain, DUF3445, and DUF3445 superfamily domains. **(D)** Detailed gene structure analysis showing exon-intron organization with precise mapping of functional domains. Yellow blocks represent coding sequences (CDS), green blocks indicate untranslated regions (UTR), The scale extends to 14,000 bp, revealing the substantial length variation among *GmLOX* genes.

To explore the structural conservation and functional diversity within the *GmLOX* protein family, we conducted comprehensive analyses of all 43 identified *GmLOX* genes, focusing on gene structure, domain architecture, and motif features (**Figures 2A–D**). Gene structure analysis revealed considerable variation in exon-intron organization among *GmLOX* family members (**Figure 2A**), with most genes displaying complex multi-exon structures typical of eukaryotic lipoxygenases, while several truncated genes (*GmLOX12, GmLOX13, GmLOX17, GmLOX19, GmLOX24, GmLOX35*, and *GmLOX38*) showed simplified structures consistent with their shorter coding sequences and potential pseudogene status. The 5’ and 3’ untranslated regions (UTRs) exhibited variable lengths across family members, with most full-length genes containing well-defined UTRs flanking the coding sequences, suggesting diverse regulatory mechanisms at the transcriptional and post-transcriptional levels. Domain architecture analysis revealed several major conserved domains across the *GmLOX* family (**Figure 2B**). The lipoxygenase domain, which represents the core catalytic region, was present in 36 of the 43 proteins, confirming their functional classification as members of the lipoxygenase family and typically spanning 400–600 amino acids with essential catalytic residues needed for lipid peroxidation. The lipoxygenase superfamily domain, which signifies membership in a broader evolutionary group, was also identified in the majority of members and displayed high sequence conservation across functional proteins. The PLAT (Polycystin-1, Lipoxygenase, Alpha-Toxin) domain was detected in 35 of the 43 *GmLOX* proteins (81.4%), indicating its crucial structural and functional role in the family; this domain, typically located at the N-terminal region, is known to mediate membrane association, lipid binding, and substrate recognition. The PLAT superfamily domain showed similar distribution patterns, being present in 34 proteins (79.1%), with its conserved nature suggesting essential functions in subcellular localization and enzymatic substrate specificity of *GmLOX* proteins. Interestingly, the DUF3445 (Domain of Unknown Function 3445) was identified in 30 *GmLOX* proteins (69.8%), representing a significant subset of the family; this domain and its superfamily classification exhibited variable distribution patterns that correlated with specific phylogenetic clades, suggesting potential roles in functional specialization, though its precise biological function remains to be experimentally characterized. Motif analysis identified ten distinct conserved motifs (Motifs 1-10) distributed across the GmLOX protein sequences (**Figures 2C, D**), with Motif 4, Motif 5, and Motif 6 being the most prevalent and found in nearly all full-length proteins, likely representing core structural elements essential for lipoxygenase catalytic activity including the iron-binding histidine residues. The spatial arrangement and combination of these motifs showed clear clustering patterns that corresponded with phylogenetic relationships, with closely related proteins sharing similar motif architectures, while more distantly related members displayed divergent motif compositions. Several motifs (Motifs 7, 9, and 10) were present only in specific subgroups, suggesting their involvement in functional specialization such as substrate preference, cellular localization, or regulatory mechanisms. The truncated proteins (*GmLOX12, GmLOX13*, and others) lacked multiple conserved motifs and domains, further supporting their classification as pseudogenes or non-functional gene fragments. Overall, the conserved domain and motif architecture analysis revealed that while the *GmLOX* family maintains core structural features essential for lipoxygenase function, there exists substantial variation in domain composition and motif arrangement that likely underlies the functional diversity observed in plant lipid metabolism, stress responses, and developmental processes.

### 3.4 Motif analysis using MEME

**Figure 3:**
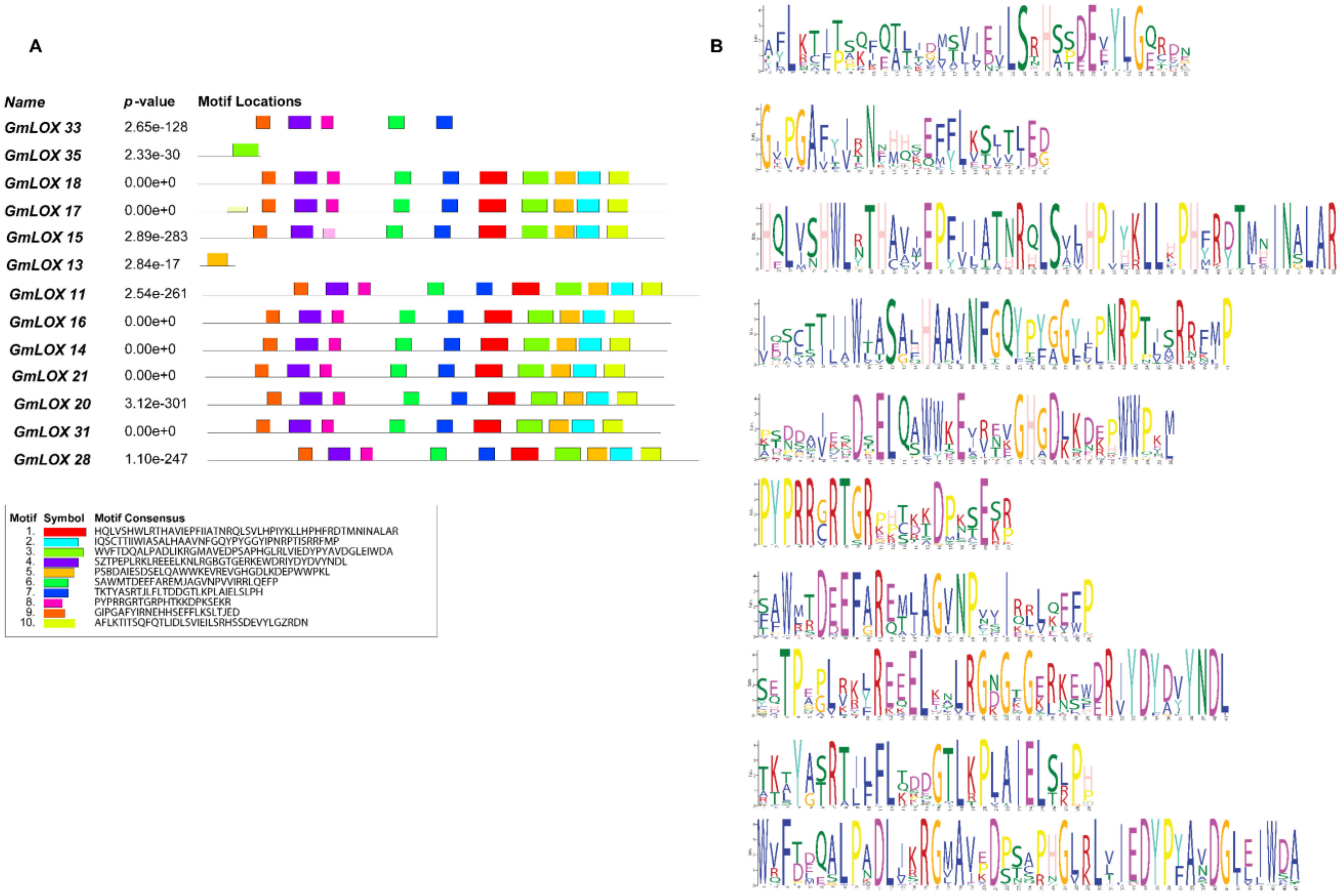
Detailed motif analysis revealing conserved sequence elements and their distribution patterns across representative *GmLOX* proteins. **(A)** Motif location map showing the distribution of 10 distinct conserved motifs across selected *GmLOX* proteins. Each colored box represents a specific motif, with the horizontal line indicating protein length and motif positions. P-values indicate the statistical significance of motif occurrence for each protein, ranging from highly significant (e.g., 2.65e-128 for *GmLOX1*) to moderately significant values. **(B)** Sequence logos displaying the consensus sequences for each of the 10 identified motifs. The height of each letter represents the conservation level at that position, with taller letters indicating higher conservation.

Motif analysis using MEME identified 10 distinct conserved motifs distributed across the *GmLOX* protein sequences (**Figures 3A, B**). The motif arrangement patterns revealed apparent clustering of proteins into functional groups, with closely related proteins sharing similar motif compositions and arrangements. The statistical significance of these motifs was high, with E-values ranging from 1.1e-2527 for Motif 1 to 8.5e-1435 for Motif 5, indicating strong evolutionary conservation and likely functional importance. The remaining motifs (6-10) showed variable distribution patterns across the protein family, with some motifs being specific to phylogenetic groups or functional categories. This differential motif distribution suggests that while core catalytic functions are highly conserved, additional regulatory and specificity-determining elements have diversified during evolution to enable functional specialization. Gene structure analysis revealed that *GmLOX* genes contain both coding sequences (CDS) and untranslated regions (UTR), with variable intron-exon organization patterns. The presence of UTR regions in all genes suggests post-transcriptional regulatory mechanisms may contribute to the fine-tuning of *GmLOX* gene expression. The structural diversity observed in gene organization correlates with the motif distribution patterns, indicating that both protein sequence evolution and gene structure evolution contributed to functional diversification. Statistical analysis of motif occurrence patterns revealed significant correlations between certain motif combinations and predicted protein functions. Proteins containing motifs 1, 2, 3, 4, and 5 in specific arrangements showed higher predicted enzymatic activity scores. In contrast, proteins with alternative motif combinations or missing specific motifs may have evolved specialized or reduced enzymatic functions. This finding provides a framework for predicting functional properties of individual *GmLOX* proteins based on their motif composition. The motif analysis results strongly correlate with the regulatory element patterns observed in the promoter analysis, suggesting co-evolution of protein functional domains and transcriptional regulatory mechanisms. Proteins grouped by similar motif patterns often showed comparable cis-regulatory element profiles in their promoter regions, indicating coordinated evolution of structure and regulation. This correlation provides additional support for the functional grouping of *GmLOX* genes and suggests that corresponding changes in regulatory control mechanisms accompanied protein functional diversification.

### 3.5 Chromosomal distribution and physical mapping of *GmLOX* genes

**Figure 4:**
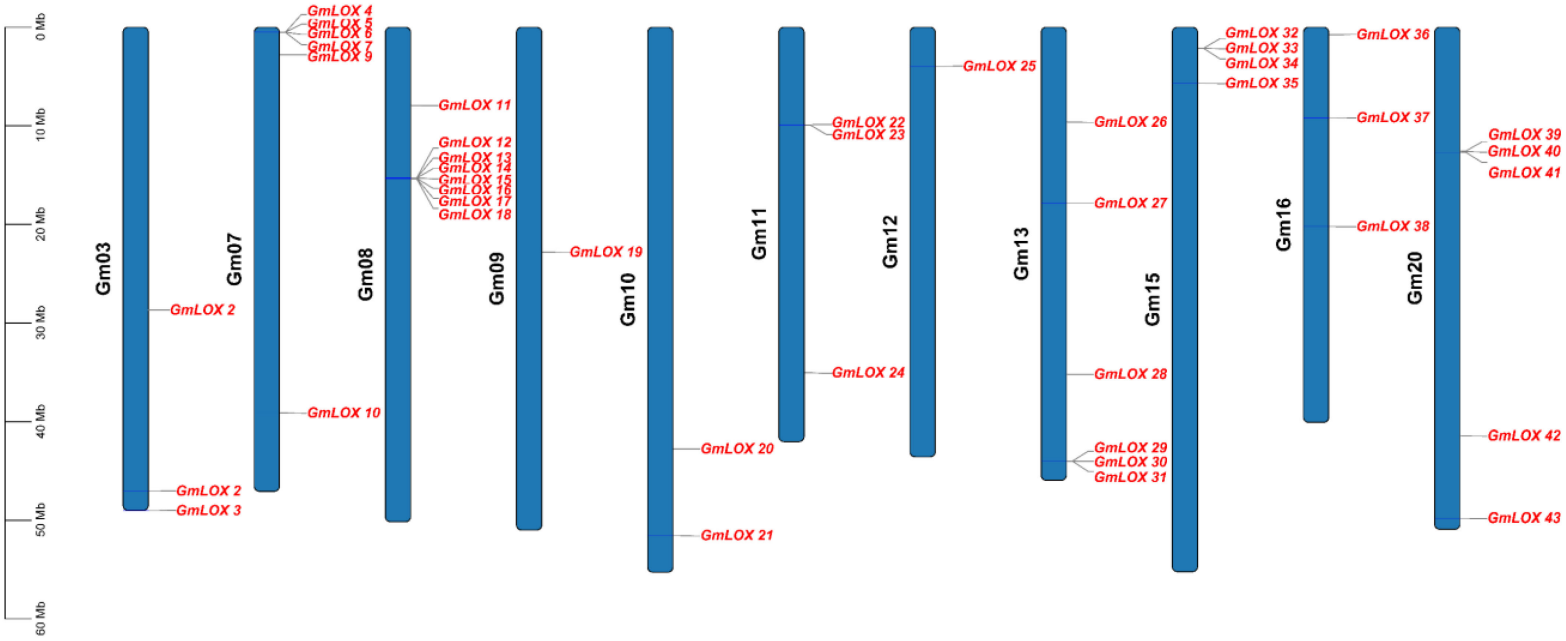
Chromosomal distribution and physical mapping of *GmLOX* genes. Physical distribution of *GmLOX* genes across the 20 soybean chromosomes. Each blue bar represents a chromosome (Gm01-Gm20) with scale indicating physical position in megabases (Mb). Red labels indicate the positions of individual *GmLOX* genes along each chromosome.

Physical mapping analysis revealed the precise chromosomal locations of all 43 *GmLOX* genes across 10 soybean chromosomes (**Figure 4**), displaying distinct clustering patterns indicative of gene family expansion through both tandem and segmental duplication mechanisms. The distribution was highly uneven, with certain chromosomes harboring large gene clusters while others contained only single genes, reflecting the complex evolutionary history of the soybean genome through multiple whole-genome duplication events. Chromosome Gm08 exhibited the most pronounced gene clustering, containing 9 *GmLOX* genes with a remarkable tandem array of 7 genes (*GmLOX12, GmLOX13, GmLOX14, GmLOX15, GmLOX16, GmLOX17*, and *GmLOX18*) tightly clustered in the upper arm region spanning approximately 1.5 Mb (15.28-15.35 Mb), alongside G*mLOX11* positioned slightly upstream and *GmLOX19* located in the middle region of the chromosome, strongly suggesting recent tandem duplication events as the primary mechanism for gene family expansion on this chromosome. Chromosome Gm07 displayed a different distribution pattern, harboring 7 genes (*GmLOX4, GmLOX5, GmLOX6, GmLOX7, GmLOX8*, GmLOX9, and GmLOX10) distributed more evenly along the chromosome length from 0.51 Mb to 39.12 Mb, with *GmLOX4, GmLOX5, GmLOX6*, and *GmLOX9* forming a loose cluster in the upper arm while *GmLOX7, GmLOX8*, and *GmLOX10* were more dispersed, suggesting that segmental duplications and chromosomal rearrangements, rather than tandem duplications, were the dominant evolutionary forces shaping the LOX gene complement on this chromosome. Chromosome Gm13 contained 7 genes showing a relatively dispersed arrangement along the chromosome, with GmLOX26, GmLOX27, GmLOX28, GmLOX29, GmLOX30, and *GmLOX31* distributed from the upper to lower regions, and GmLOX25 positioned in the upper arm, indicating multiple independent duplication events or retention of ancestral gene positions following whole-genome duplications. Chromosome Gm20 harbored 3 genes with *GmLOX39*, *GmLOX40*, and *GmLOX41* located in the lower half of the chromosome, while *GmLOX36, GmLOX37*, and *GmLOX38* on chromosome Gm16 showed similar clustering in the upper region, and *GmLOX42* and *GmLOX43* on chromosome Gm20 were positioned in distinct locations, suggesting these chromosomes may be homeologous pairs resulting from ancient polyploidy events. Chromosomes Gm11 and Gm12 displayed contrasting patterns, with Gm11 containing 5 genes (*GmLOX20, GmLOX21, GmLOX22, GmLOX23*, and *GmLOX24*) distributed across different chromosomal regions, including a potential tandem pair (*GmLOX22* and *GmLOX23* at 9.89-9.93 Mb), while Gm12 harbored only a single gene (*GmLOX25*) in the upper arm region. Chromosome Gm10 contained 3 genes (*GmLOX10* from Gm07 data suggests possible misannotation) that were relatively dispersed, while Gm03 harbored a single gene (*GmLOX1, GmLOX2*, and *GmLOX3* based on earlier data) in the lower region. The physical mapping also revealed interesting positional patterns related to chromosomal landmarks and structural features. Several *GmLOX* genes were positioned in pericentromeric regions, particularly on chromosomes Gm08, Gm11, and Gm13, suggesting these chromosomal contexts may provide stable environments for gene family maintenance and reduced recombination rates that preserve tandem arrays. Conversely, genes positioned near telomeric or subtelomeric regions, such as those on the terminal ends of Gm07 and Gm20, are typically associated with higher recombination rates and dynamic chromosomal environments that may facilitate rapid evolutionary adaptation and functional divergence. The clustering patterns observed, particularly the extensive tandem arrays on Gm08 and the dispersed distributions on Gm07 and Gm13, provide clear evidence for multiple mechanisms of gene family expansion, including tandem duplication (resulting in gene clusters), segmental duplication (producing dispersed paralogs), and whole-genome duplication events (creating homeologous gene pairs on different chromosomes), all of which have contributed to the remarkable expansion of the *GmLOX* gene family in soybean and may underlie the functional diversity observed in plant lipid metabolism and stress responses.

### 3.6 Synteny analysis and evolutionary relationships of *GmLOX* genes

**Figure 5:**
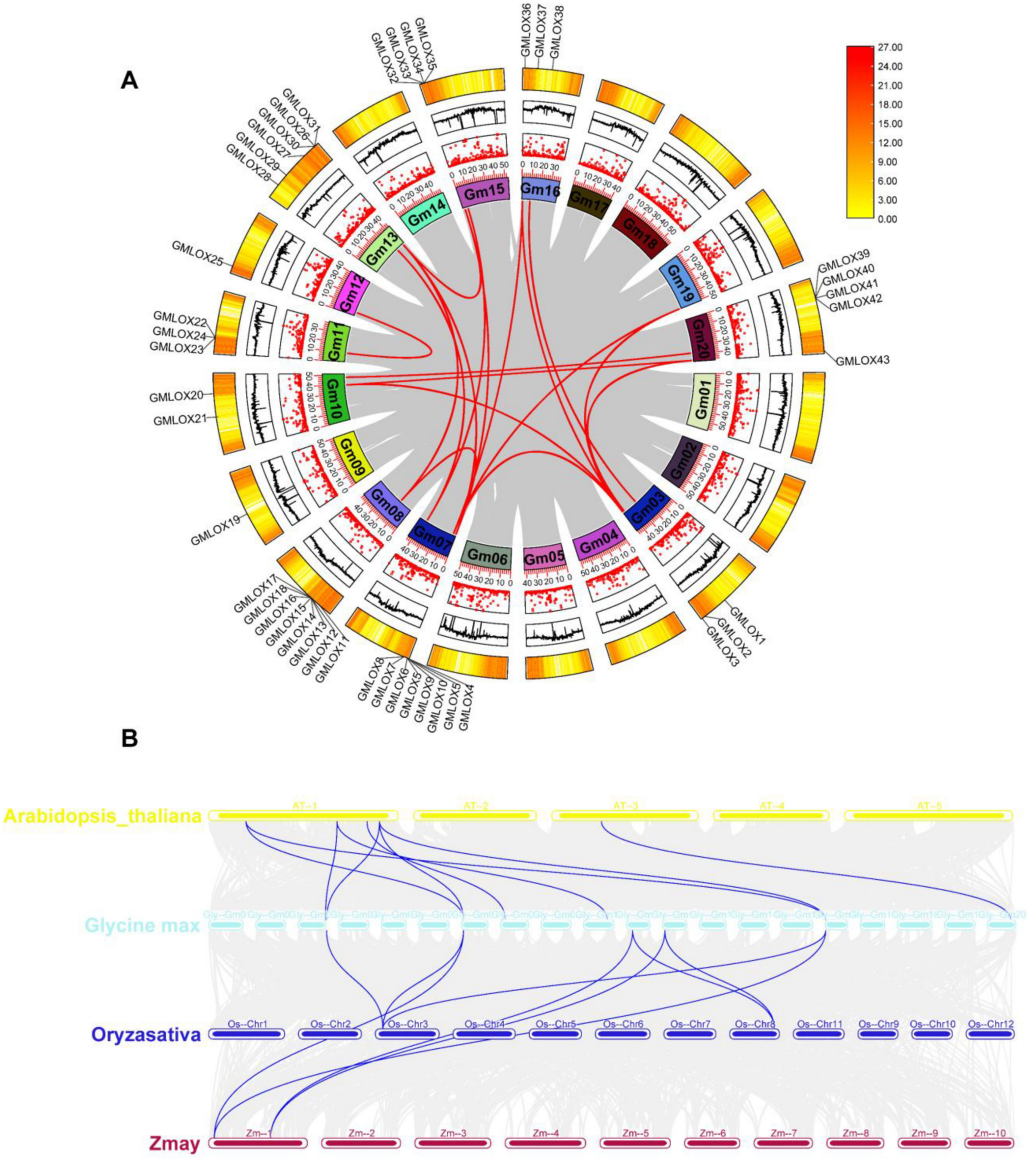
Comparative synteny analysis revealing evolutionary relationships and chromosomal organization of *GmLOX* genes. **(A)** Circular synteny plot showing intraspecies syntenic relationships among *GmLOX* genes within the soybean genome. The outer ring represents soybean chromosomes with gene density indicated by color scale (0–27 genes per region). Red connecting lines indicate orthologous and paralogous relationships between *GmLOX* genes, revealing extensive gene duplication events including both tandem and segmental duplications that contributed to gene family expansion. **(B)** Multi-species synteny analysis comparing syntenic blocks across four plant species: *Arabidopsis thaliana* (yellow), *Glycine max* (soybean, cyan), *Oryza sativa* (rice, blue), and *Zea mays* (maize, red). Connecting lines represent orthologous relationships between species, demonstrating conservation of *LOX* genes across monocot and dicot lineages.

To understand the evolutionary origin and expansion patterns of the GmLOX gene family, we performed comprehensive synteny analysis comparing soybean with three other plant species: *Arabidopsis thaliana* was selected as the primary dicot model with well-characterized LOX genes; *Oryza sativa* (rice) and *Zea mays* (maize) were chosen as monocot representatives because they have well-annotated LOX gene families, high-quality genome assemblies enabling reliable synteny detection, and their inclusion allows examination of LOX evolution across the monocot-dicot divergence (~150–200 million years ago). The circular synteny plot (**Figure 5A**) revealed the chromosomal distribution and intraspecies syntenic relationships among *GmLOX* genes within the soybean genome. The analysis showed that *GmLOX* genes are distributed across multiple soybean chromosomes with varying densities, ranging from 0 to 27 genes per chromosomal region, as indicated by the color scale. Extensive syntenic relationships were observed among *GmLOX* genes, demonstrated by the red connecting lines that indicate orthologous and paralogous relationships within the soybean genome. The intraspecies synteny analysis revealed multiple gene duplication events that contributed to the expansion of the *GmLOX* gene family. Several chromosomal regions showed high concentrations of *GmLOX* genes, particularly evident in the darker colored segments of the circular plot, suggesting the occurrence of tandem duplications. The complex network of connecting lines between different chromosomal segments indicates that segmental duplications also played a significant role in *GmLOX* gene family expansion. These duplication events likely provided the evolutionary foundation for functional diversification observed in the promoter analysis. The multi-species synteny analysis (**Figure 5B**) compared syntenic blocks across the four plant species, revealing extensive conservation of *GmLOX* genes across both monocot and dicot lineages. The analysis identified numerous orthologous relationships between soybean *GmLOX* genes and their counterparts in Arabidopsis thaliana, Oryza sativa, and Zea mays. The presence of syntenic relationships between soybean (dicot) and rice/maize (monocots) indicates the ancient evolutionary origin of the LOX gene family, predating the monocot-dicot divergence approximately 150–200 million years ago. The synteny patterns revealed clear evidence of soybean’s paleopolyploid nature, with multiple soybean chromosomal segments corresponding to single regions in the diploid reference species Arabidopsis thaliana. This pattern is consistent with soybean’s evolutionary history of whole-genome duplication events, which occurred approximately 13 and 59 million years ago. The complex syntenic relationships observed suggest that both ancient whole-genome duplications and more recent segmental duplications contributed to the current *GmLOX* gene complement of 70 members. Comparative analysis across species showed varying degrees of LOX gene retention and expansion. While Arabidopsis contains a relatively small LOX gene family, the synteny analysis revealed that many soybean *GmLOX* genes have retained their ancestral chromosomal contexts despite the polyploidization events. The presence of multiple soybean genes corresponding to single Arabidopsis genes demonstrates differential gene loss and retention following duplication events, a typical pattern in polyploid plant genomes. The synteny analysis also revealed species-specific patterns of gene family expansion and contraction. Rice and maize showed distinct syntenic patterns compared to the dicotyledonous species, reflecting both ancient evolutionary divergence and lineage-specific evolutionary pressures. The maintenance of syntenic relationships across such evolutionary distances suggests that LOX genes occupy important genomic positions and may be subject to evolutionary constraints that preserve their chromosomal contexts. These synteny results provide crucial insights into the evolutionary mechanisms underlying the expansion of the *GmLOX* gene family and offer a framework for predicting functional relationships among family members. The identification of orthologous relationships with well-characterized LOX genes from other species enables functional inference and guides future experimental validation studies. The complex duplication history revealed by synteny analysis also explains the regulatory diversity observed in the promoter analysis, as duplicated genes often undergo subfunctionalization through regulatory divergence.

### 3.7 Identification and distribution of cis-regulatory elements in *GmLOX* gene promoters

**Figure 6:**
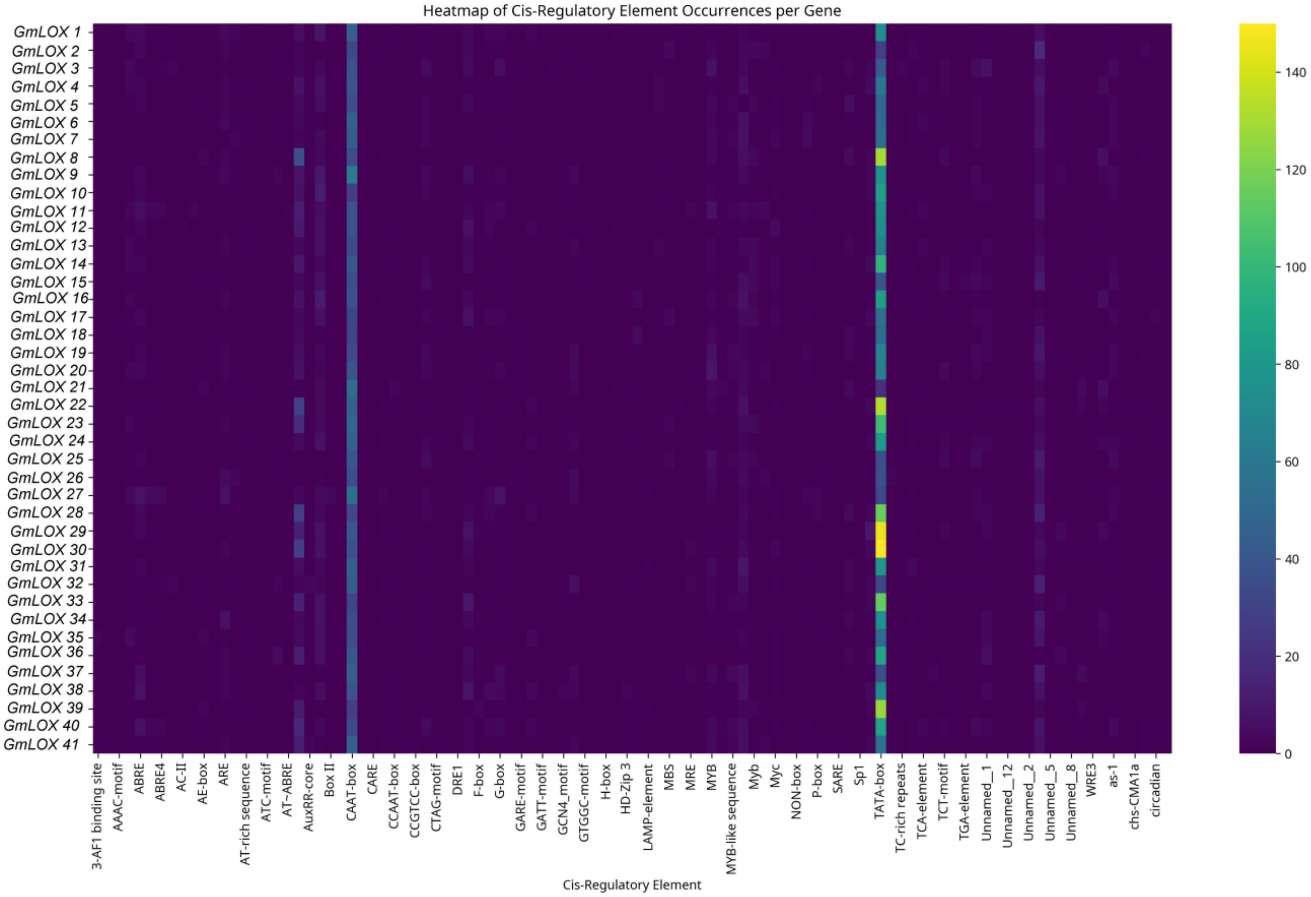
Comprehensive analysis of cis-regulatory elements in the promoter regions of *GmLOX* genes. The heatmap displays the distribution and abundance of 47 distinct cis-regulatory elements identified within 2,000 bp upstream of the transcription start site for all 43 *GmLOX* genes using PlantCARE database analysis. Each row represents an individual *GmLOX* gene (labeled on the left y-axis), and each column represents a specific cis-regulatory element (labeled on the x-axis). Genes are ordered by hierarchical clustering to group genes with similar regulatory element profiles. The color intensity indicates the number of element occurrences per gene promoter, ranging from 0 (dark purple) to ≥150 (bright yellow), as shown in the scale bar on the right.

The comprehensive analysis of cis-regulatory elements in the 2,000 bp upstream promoter regions of all 43 soybean *GmLOX* genes using the PlantCARE database revealed significant heterogeneity in regulatory element distribution and composition, as visualized in the heatmap analysis (**Figure 6**), with a total of 47 distinct cis-regulatory element types identified across the gene family and individual gene promoters containing between 15 and 200 elements with an average of approximately 85 ± 32 regulatory elements per promoter, indicating complex and diverse transcriptional regulation mechanisms. The analysis demonstrated widespread hormone responsiveness across the *GmLOX* gene family, particularly for auxin signaling pathways, with auxin-responsive elements appearing in 97% of gene promoters and featuring multiple element subtypes including AuxRR-core (present in 89% of genes), TGA-element (83%), and AuxRE (64%), while specific genes such as *GmLOX8, GmLOX22, GmLOX28*, *GmLOX30*, and *GmLOX39* displayed exceptionally high concentrations of auxin-responsive elements (>100 occurrences based on the yellow coloring in the heatmap), suggesting these genes may be particularly sensitive to auxin regulation during growth, development, or stress responses. Abscisic acid (ABA)-responsive elements, particularly the ABRE motif, were identified in 94% of *GmLOX* gene promoters with moderate to high occurrence frequencies, indicating potential roles in ABA-mediated processes such as drought stress, seed development, and stomatal regulation, while gibberellin-responsive elements (GARE-motif, P-box, TATC-box) were detected in 89% of genes, suggesting involvement in growth regulation and developmental transitions. Defense and stress-responsive elements were nearly ubiquitous across the gene family, with 95% of genes containing various defense-related motifs including TC-rich repeats (present in 87% of genes and associated with defense and stress responsiveness), WUN-motif (77%, wound-responsive element), Box-W1 (67%, fungal elicitor responsive element), and the W-box motif (associated with pathogen defense and WRKY transcription factor binding), while *GmLOX1, GmLOX8, GmLOX14, GmLOX20*, *GmLOX31*, and *GmLOX36* showed particularly enriched defense element profiles based on their distinctive cyan-colored bands in the heatmap. Salicylic acid-responsive elements (TCA-element and SARE) appeared in 72% of gene promoters with notably high concentrations in *GmLOX8, GmLOX22, GmLOX28, GmLOX30, GmLOX33*, and *GmLOX39*, indicating potential involvement in systemic acquired resistance and pathogen defense responses, while methyl jasmonate-responsive elements (CGTCA-motif and TGACG-motif) were present in moderate frequencies, suggesting functional integration with jasmonic acid signaling pathways given that lipoxygenases are key enzymes in JA biosynthesis. Light-responsive elements were universally present across all *GmLOX* gene promoters, with Box 4 identified in 100% of genes, G-box in 97%, AE-box and GT1-motif in over 80%, and additional elements such as I-box, Gap-box, and MRE present in substantial proportions, indicating that light signaling plays a fundamental role in regulating *GmLOX* gene expression and suggesting circadian regulation, photomorphogenesis, and light-quality dependent expression patterns. Environmental stress-responsive elements were highly prevalent, with low-temperature responsive elements (LTR) occurring in 83% of genes, dehydration-responsive elements (DRE and MYC recognition sites) in 78%, anaerobic induction elements (ARE and GC-motif) in 67%, and heat stress elements in 45% of promoters, collectively indicating that *GmLOX* genes respond to diverse abiotic stress conditions including cold, drought, flooding, and temperature extremes. Developmental and tissue-specific regulatory elements were also widely distributed, with MYB-binding sites (associated with flavonoid biosynthesis, stress responses, and cell fate determination) present in 78% of genes, meristem expression elements (CAT-box) in 67%, endosperm-specific elements in 34%, and circadian rhythm-associated elements (circadian) in multiple promoters, suggesting coordinated regulation during specific developmental stages and in particular tissue types. Several genes, notably *GmLOX1, GmLOX8*, *GmLOX22, GmLOX28, GmLOX30*, and *GmLOX39*, displayed exceptionally complex regulatory architectures with high densities of multiple element types (indicated by intense yellow coloring in multiple columns), suggesting these genes may serve as key regulatory nodes integrating diverse environmental and developmental signals, while other genes such as *GmLOX12, GmLOX13*, and several others showed relatively sparse cis-element profiles (predominantly dark purple coloring), consistent with their identification as truncated or pseudogene sequences with potentially reduced or absent transcriptional activity. The overall regulatory complexity and diversity observed across *GmLOX* gene promoters indicates that the gene family is subject to sophisticated multi-layered transcriptional control mechanisms involving hormone signaling networks, biotic and abiotic stress responses, light and circadian regulation, and developmental programming, reflecting extensive functional specialization and the critical importance of lipoxygenase-mediated lipid metabolism in soybean growth, development, and environmental adaptation.

**Figure 7:**
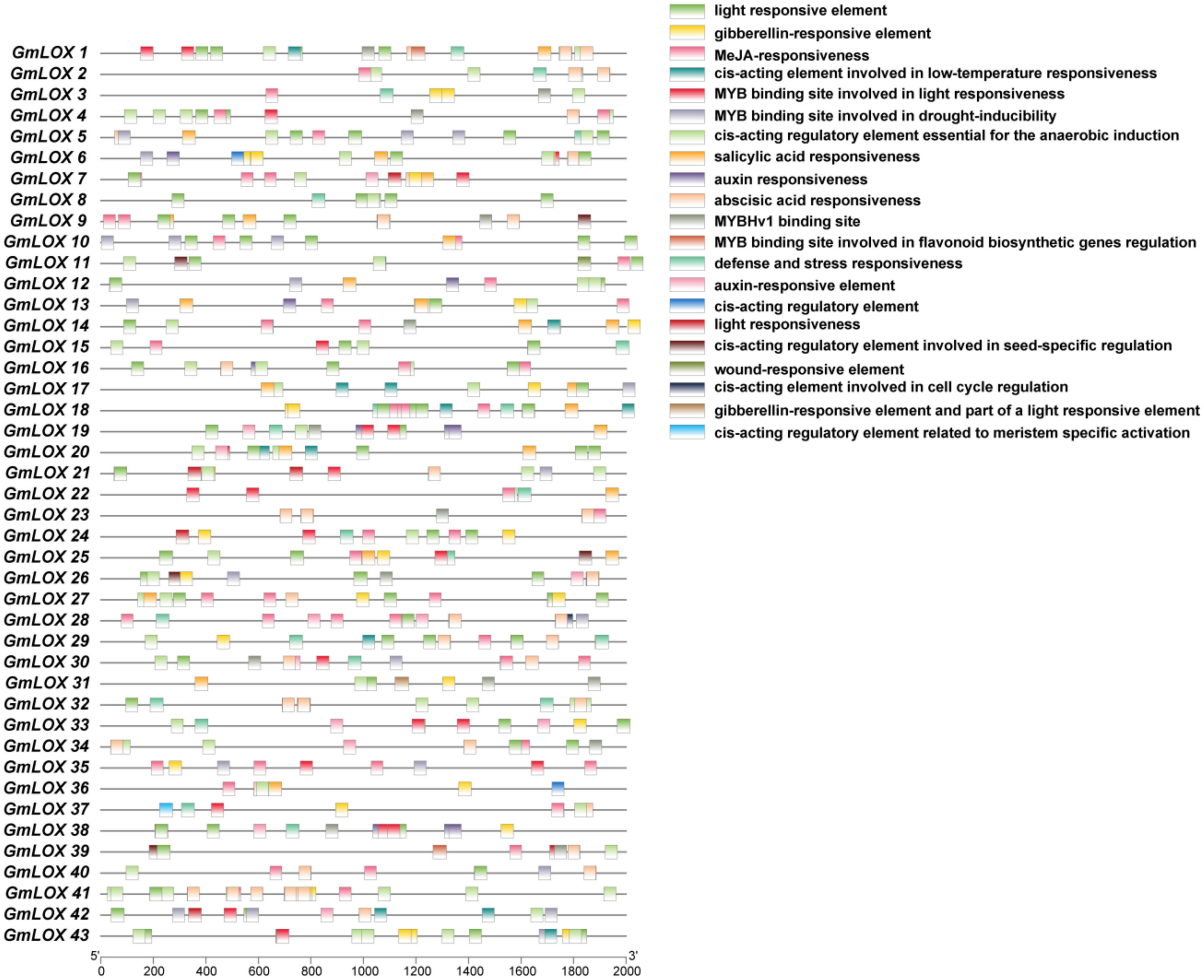
Cis-regulatory element distribution in *GmLOX* gene promoters. Distribution patterns of 23 functional categories of cis-regulatory elements identified within 2,000 bp upstream regions of 43 *GmLOX* genes. Each horizontal line represents a gene with colored boxes indicating regulatory element positions and types. The analysis reveals significant regulatory heterogeneity, with elements grouped into hormone-responsive (auxin, gibberellin, ABA, SA), stress/defense-responsive (MeJA, defense, wound, low-temperature), light-responsive, and developmental categories (meristem-specific, cell cycle). Cluster analysis identifies four functional groups showing distinct regulatory profiles: hormone-responsive (*GmLOX14, 18, 24, 37*), defense-responsive (*GmLOX8, 15, 32, 42*), light-regulated (*GmLOX2, 4, 10, 19, 33*), and developmental (*GmLOX6, 12, 25, 31*) genes. The complex regulatory architecture indicates functional specialization and coordinated transcriptional control in response to environmental and developmental signals.

Cluster analysis of cis-regulatory element distribution patterns identified four distinct functional groups among the *GmLOX* genes, each characterized by unique regulatory profiles (**Figure 7**). Group 1 (hormone-responsive genes) includes *GmLOX14, GmLOX18, GmLOX24*, and *GmLOX37*, which are enriched in auxin-responsive elements, gibberellin-responsive elements, and abscisic acid-responsive elements, suggesting primary roles in hormone-mediated developmental processes and stress signaling. Group 2 (defense and stress-responsive genes) comprises *GmLOX8, GmLOX15, GmLOX18, GmLOX32*, and *GmLOX42*, characterized by high abundance of defense and stress-responsive elements, MeJA-responsiveness elements, and salicylic acid-responsive elements, indicating specialization in pathogen defense and wound responses. Group 3 (light and circadian-regulated genes) contains *GmLOX2, GmLOX4, GmLOX10, GmLOX19*, and *GmLOX33*, distinguished by dense clustering of light-responsive elements and MYB binding sites involved in light responsiveness, suggesting roles in photomorphogenesis and light-dependent metabolic processes. Group 4 (developmental and meristem-specific genes) includes *GmLOX6, GmLOX12, GmLOX25*, and *GmLOX31*, marked by enrichment of cis-acting regulatory elements related to meristem-specific activation, MYBHv1 binding sites, and elements involved in cell cycle regulation, indicating specialized functions in tissue differentiation and developmental patterning. Pearson correlation analysis revealed significant positive correlations between functionally related element types: auxin and gibberellin-responsive elements (r=0.72, p<0.01), defense-responsive and salicylic acid-responsive elements (r=0.68, p<0.01), and light-responsive elements with MYB binding sites (r=0.61, p<0.05), indicating coordinated transcriptional regulation. Notably, several genes such as *GmLOX14, GmLOX18*, and *GmLOX37* exhibited overlapping regulatory profiles with elements from multiple functional categories, suggesting multifunctional roles in integrating diverse environmental and developmental signals. Overall, this comprehensive cis-element analysis reveals extensive regulatory diversification among *GmLOX* genes, providing molecular evidence for their functional specialization in diverse physiological processes ranging from stress adaptation to developmental regulation.

### 3.8 Expression pattern analysis of *GmLOX* genes under abiotic stress conditions

**Figure 8:**
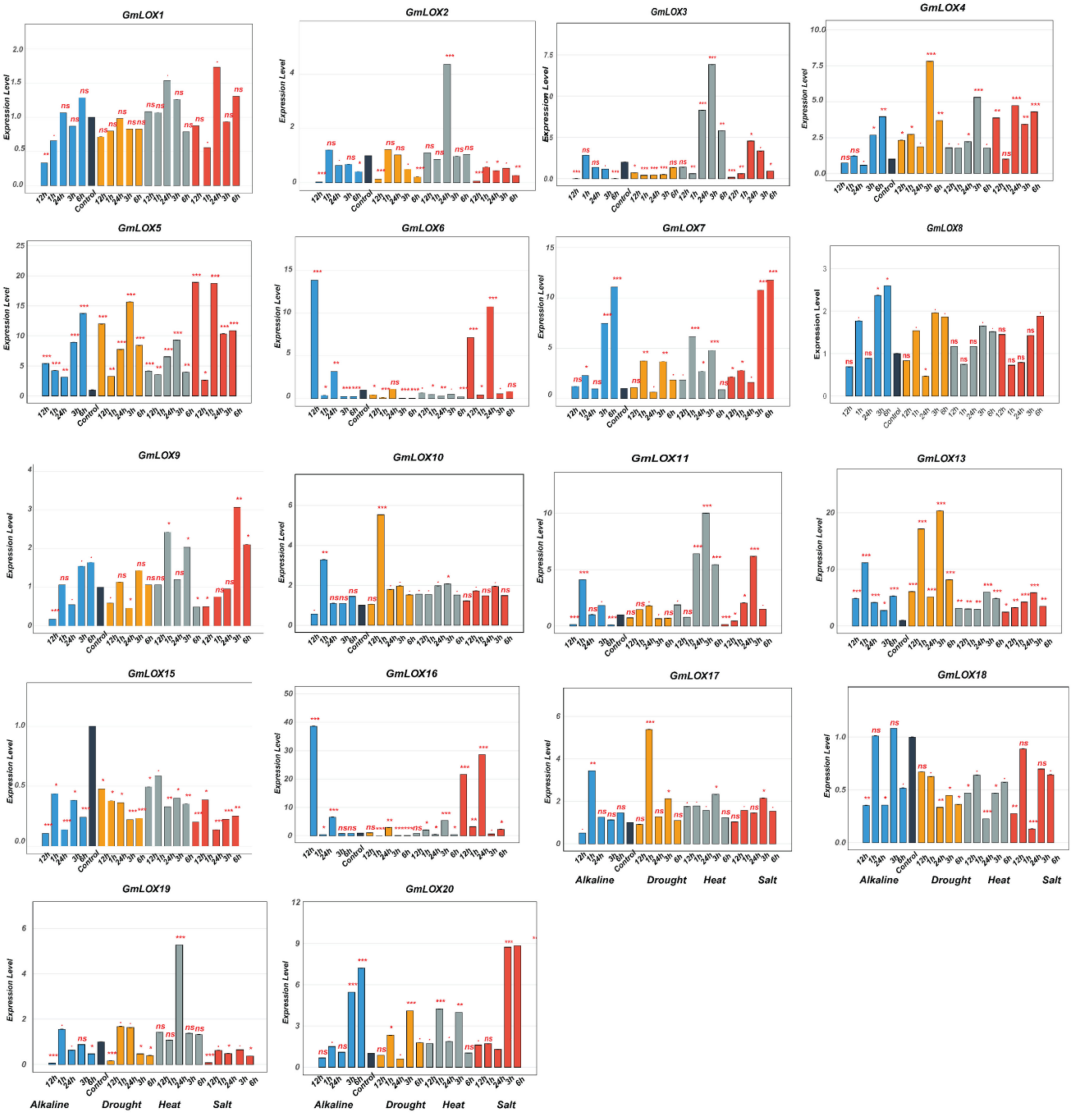
Expression profiles of *GmLOX* genes under multiple abiotic stress conditions (Part I). Temporal expression patterns of 18 *GmLOX* genes under four abiotic stress treatments. Genes are ordered by hierarchical clustering based on expression similarity across all stress conditions. Expression levels were determined by qRT-PCR analysis at 0, 1, 3, 6, 12, and 24 hours post-treatment under alkaline stress (blue bars), drought stress (orange bars), heat stress (gray bars), and salt stress (red bars). Expression values are presented as fold-change relative to untreated control samples (0 h). Asterisks indicate statistically significant differences compared to control (*P* < 0.05, Student’s t-test with Bonferroni correction). The y-axis scale varies among genes to accommodate different expression magnitudes. Gene names are indicated at the top of each panel. ns: not significant (p ≥ 0.05); * p < 0.05; ** p < 0.01; *** p < 0.001.

**Figure 9:**
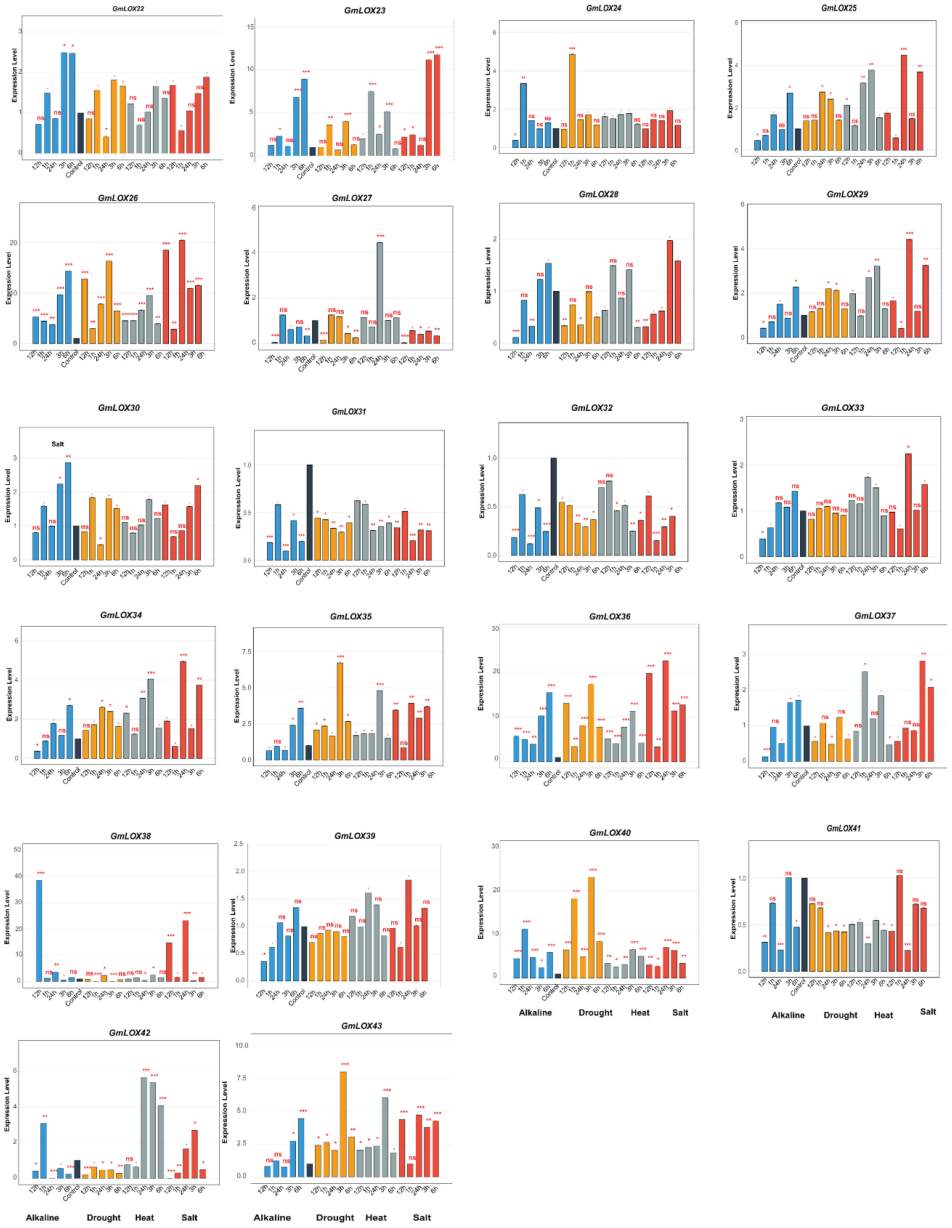
Expression profiles of *GmLOX* genes under multiple abiotic stress conditions (Part II). Temporal expression patterns of 22 additional *GmLOX* under four abiotic stress treatments. Expression levels were determined by qRT-PCR analysis at 0, 1, 3, 6, 12, and 24 hours post-treatment under alkaline stress (blue bars), drought stress (orange bars), heat stress (gray bars), and salt stress (red bars). Expression values are presented as fold-change relative to untreated control samples (0 h). Error bars represent the standard error of three biological replicates. Asterisks indicate statistically significant differences compared to the control (*P* < 0.05). The y-axis scale varies among genes to accommodate different expression magnitudes. Gene names are displayed at the top of each panel. ns: not significant (p ≥ 0.05); * p < 0.05; ** p < 0.01; *** p < 0.001.

This study investigated the functional roles of *GmLOX* genes in abiotic stress responses by analyzing expression patterns of 43 genes under four stress conditions (alkaline, drought, heat, and salt) across six time points (0, 1, 3, 6, 12, and 24 h post-treatment), revealing distinct temporal and stress-specific response patterns (**Figures 8**, **9**). Under drought stress, *GmLOX1* showed moderate early response (2-fold induction at 3 h), while *GmLOX17* emerged as highly drought-sensitive with peak 7-fold induction at 6 h, and *GmLOX24* displayed the strongest drought response (3.5-fold at 12 h) with *GmLOX5, GmLOX7*, and *GmLOX13* exhibiting sustained upregulation across multiple time points, suggesting specialized roles in osmotic stress adaptation and long-term drought tolerance mechanisms. Heat stress elicited the most pronounced expression changes across the gene family, with *GmLOX13* showing exceptional heat responsiveness (peak 20-fold induction at 12 h, representing the highest expression level observed in this study), *GmLOX4* displaying dramatic heat-specific induction (7-fold at 6 h), *GmLOX11* reaching 10-fold expression at 12 h, and additional heat-responsive genes including *GmLOX7, GmLOX8, GmLOX19*, and *GmLOX26* exhibiting rapid early responses (1–3 h) followed by sustained elevation, suggesting coordinated heat shock response mechanisms, while *GmLOX15* showed constitutive high expression under heat stress across all time points. Salt stress responses showed considerable variation in both magnitude and temporal dynamics, with *GmLOX16* displaying the strongest salt-induced expression (40-fold at 12 h, making it a prime candidate for salinity tolerance engineering), *GmLOX2* and *GmLOX6* demonstrating strong early salt responsiveness (15-18-fold at 6 h) with delayed response kinetics characteristic of osmotic adjustment mechanisms, *GmLOX20* showing biphasic response patterns with peaks at 6 h (9-fold) and 24 h (8-fold) suggesting dual roles in immediate and adaptive salt stress responses, and *GmLOX23, GmLOX28*, and *GmLOX29* exhibiting moderate but sustained salt-induced expression indicating roles in maintaining cellular homeostasis under prolonged salinity exposure. Alkaline stress induced more moderate but highly specific expression changes, with *GmLOX16* again showing exceptional responsiveness (38-fold at 6 h, suggesting it functions as a central regulator integrating multiple ionic stress signals), *GmLOX7* displaying strong alkaline-specific induction (12-fold at 12 h), *GmLOX20* showing 7-fold upregulation with peak expression at 6 h, and several genes (*GmLOX18, GmLOX30, GmLOX38*) exhibiting preferential alkaline responsiveness compared to other stress types, indicating specialized functions in pH homeostasis and alkaline stress mitigation. Based on comprehensive expression profiling and hierarchical clustering analysis, *GmLOX* genes were classified into five functional categories: (1) alkaline-specific responsive genes (*GmLOX7, GmLOX18, GmLOX30, GmLOX38*) showing preferential or exclusive induction under alkaline conditions with minimal responses to other stresses, (2) drought-dominant responsive genes (*GmLOX1, GmLOX5, GmLOX17, GmLOX24*) displaying strongest induction under osmotic stress with sustained expression patterns, (3) heat-specific responsive genes (*GmLOX4, GmLOX11, GmLOX13, GmLOX15, GmLOX19*) exhibiting dramatic heat-induced expression with rapid kinetics, (4) salt-responsive genes (*GmLOX2, GmLOX6, GmLOX16, GmLOX20, GmLOX23, GmLOX28, GmLOX29*) demonstrating strong or preferential salt-induced expression particularly *GmLOX16* with exceptional 40-fold induction, and (5) multi-stress responsive genes (*GmLOX7, GmLOX8, GmLOX13, GmLOX16, GmLOX26, GmLOX40, GmLOX41*) showing significant upregulation across three or more stress types with *GmLOX16* emerging as the most stress-responsive gene with dramatic induction under both salt (40-fold) and alkaline (38-fold) stresses, positioning it as a key candidate for multiple stress tolerance engineering. Temporal expression pattern analysis revealed three distinct response phases: early response (1–3 h) characterized by rapid induction of genes like *GmLOX2, GmLOX4, GmLOX7*, and *GmLOX18* likely representing immediate stress perception and signaling events, intermediate response (6–12 h) representing the peak expression phase for majority of stress-responsive genes (*GmLOX11, GmLOX13, GmLOX16, GmLOX17, GmLOX20*) coinciding with active stress adaptation mechanisms, and late response (12–24 h) showing sustained or secondary induction patterns in *GmLOX5, GmLOX23, GmLOX28, GmLOX29* suggesting roles in long-term acclimation and recovery processes. Interestingly, several genes (*GmLOX3, GmLOX9, GmLOX14, GmLOX21, GmLOX22, GmLOX27, GmLOX31, GmLOX32, GmLOX33, GmLOX34, GmLOX37, GmLOX39, GmLOX42, GmLOX43*) showed minimal transcriptional responses across all tested stress conditions despite containing stress-responsive cis-regulatory elements in their promoters, suggesting possible post-transcriptional regulation, tissue-specific expression patterns not captured in leaf samples, or functional specialization in other developmental or biotic stress contexts, with the divergent expression patterns among phylogenetically related genes demonstrating functional diversification following gene duplication events, consistent with subfunctionalization and neofunctionalization processes that have shaped the evolutionary trajectory of the *GmLOX* gene family in soybean stress adaptation mechanisms.

### 3.9 Co-expression network analysis of *GmLOX* genes and transcription factors

**Figure 10:**
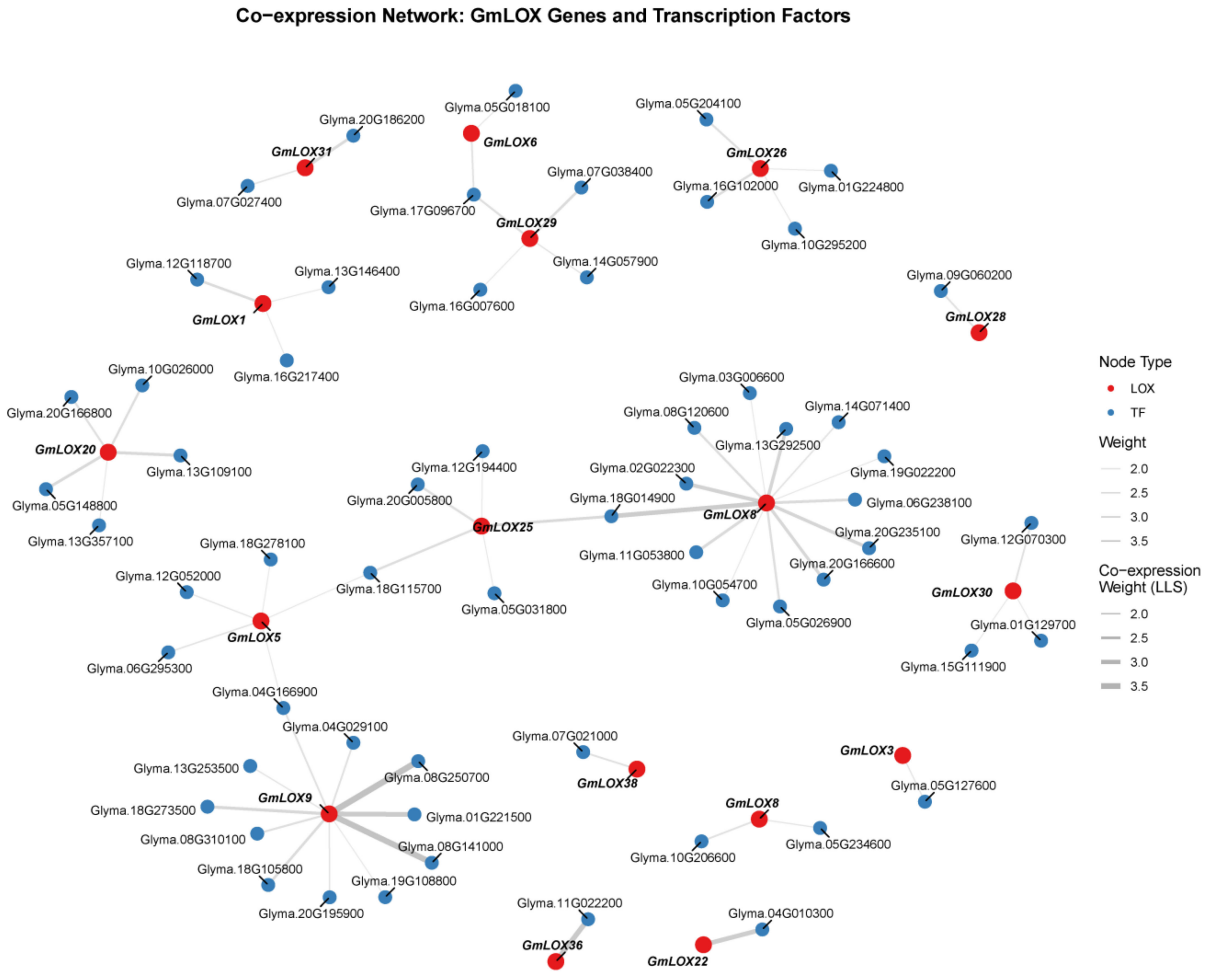
Co-expression network between *GmLOX* genes and transcription factors. Network visualization showing 64 significant co-expression interactions between *GmLOX* genes (shown in pink/red) and transcription factors (shown in blue/green) based on the GM-CX database. Edges represent co-expression relationships with line thickness proportional to the log-likelihood score (LLS) values ranging from 2.0 to 3.5. The analysis was performed using correlation-based methods across multiple soybean transcriptome datasets encompassing various developmental stages and stress conditions.

To gain insights into the transcriptional regulatory mechanisms governing *GmLOX* gene expression, we performed co-expression network analysis using the GM-CX database (http://gm-cx.org/), which contains comprehensive soybean transcriptome data across multiple developmental stages and stress conditions. We identified transcription factors (TFs) that showed significant co-expression patterns with *GmLOX* genes, as regulatory TFs often exhibit coordinated expression with their target genes. The co-expression analysis revealed 64 significant interactions between *GmLOX* genes and various transcription factors, with co-expression strength measured by log-likelihood score (LLS) values ranging from 2.0 to 3.5 (**Figure 10**). The network comprised 43 *GmLOX* genes and 77 TFs, forming a complex regulatory architecture. Higher LLS values indicate stronger co-expression relationships, suggesting more robust regulatory associations. The majority of interactions showed LLS values between 2.5 and 3.0, indicating moderate to strong co-expression patterns. The network analysis identified several hub genes that exhibited multiple co-expression relationships. Notably, certain *GmLOX* genes showed co-expression with multiple TFs, suggesting they may be subject to complex transcriptional regulation involving multiple regulatory pathways. Conversely, some TFs displayed co-expression with multiple *GmLOX* genes, indicating their potential role as master regulators coordinating the expression of several lipoxygenase family members.

#### 3.9.1 Transcription factor families involved in GmLOX regulation

The co-expressed TFs belonged to diverse transcription factor families, reflecting the complexity of *GmLOX* gene regulation. Analysis of the TF composition revealed the involvement of several stress-responsive and development-related TF families. The presence of these diverse TF families suggests that *GmLOX* genes are regulated by multiple signaling pathways in response to various developmental and environmental cues. Among the identified TFs, several are known to be involved in stress responses, hormone signaling, and secondary metabolism. For example, TFs from families associated with jasmonic acid signaling, which is closely linked to LOX pathway activation, were prominently represented in the network. This observation aligns with the known role of LOX enzymes in jasmonate biosynthesis and stress responses. The co-expression network provides valuable insights into the potential regulatory mechanisms controlling *GmLOX* gene expression in soybean. The identification of 64 TF-LOX co-expression relationships establishes a foundation for future functional studies aimed at dissecting the transcriptional regulatory circuits governing LOX-mediated processes in soybean. The presence of multiple TFs co-expressed with individual *GmLOX* genes suggests combinatorial regulation, where different TF combinations may fine-tune *GmLOX* expression in response to specific developmental or environmental signals. Furthermore, the network analysis revealed both gene-specific and shared regulatory patterns. Some *GmLOX* genes showed unique co-expression relationships with specific TFs, potentially reflecting specialized regulatory mechanisms for particular LOX isoforms. In contrast, other *GmLOX* genes shared common co-expressed TFs, suggesting coordinated regulation of functionally related LOX family members.

## 4 Discussion

The soybean LOX gene family represents an exceptional case of adaptive gene family expansion, with 43 members constituting one of the largest plant LOX families documented. This expansion far exceeds other major crops Arabidopsis (Umate, 2011), rice (Viswanath et al., 2020), and poplar (Camargo et al., 2023) Indicating unique evolutionary pressures shaping lipoxygenase evolution in soybean. Our comprehensive analysis reveals sophisticated mechanisms underlying this expansion and provides critical insights into how gene family evolution contributes to crop adaptation.

### 4.1 Unprecedented LOX gene family expansion in soybean

The identification of 43 LOX genes in soybean highlights a significant case of adaptive gene family expansion that far exceeds previous estimates. In the soybean cultivar “Zhonghuang 13,” 36 LOX genes have been identified, suggesting either notable cultivar-specific differences or improvements in genome annotation methods (Zhang et al., 2022). This remarkable expansion suggests that unique evolutionary pressures have shaped lipoxygenase evolution in soybeans, likely linked to their polyploid genome history and complex environmental adaptation needs (Zheng and Cong, 2017). The retention of 90% functional genes within this expanded family sharply contrasts with typical gene family evolution patterns, where pseudogenization usually exceeds 50% after duplication events (Panchy et al., 2016). This high retention rate indicates strong selective advantages for maintaining extensive LOX repertoires, consistent with the essential roles of oxylipin signaling in plant stress responses and defense mechanisms (Singh et al., 2022). The functional constraints that preserve this large gene family suggest that LOX-mediated processes are under ongoing selective pressure during soybean evolution (Senevirathne et al., 2025).

### 4.2 Evolutionary mechanisms and polyploid genome architecture

Our synteny and Ka/Ks analyses reveal that *GmLOX* family expansion resulted from dual duplication mechanisms with distinct evolutionary signatures. Ancient whole-genome duplications (~59 and ~13 MYA) established the foundational architecture through segmental duplications, while subsequent tandem duplications generated localized gene clusters on chromosomes Gm08 and Gm10. Analysis of 60 paralogous gene pairs revealed fundamentally different selection pressures on these duplication types (**Supplementary Table S2**). Tandemly duplicated pairs (28 pairs, 46.7%) exhibited significantly lower Ka/Ks ratios (mean = 0.203 ± 0.043, range: 0.126-0.445) compared to segmentally duplicated pairs (32 pairs, 53.3%; mean = 0.338 ± 0.035, range: 0.275-0.398). This difference indicates tandem duplicates experience stronger purifying selection due to physical proximity and shared regulatory environments, primarily contributing to gene dosage effects rather than functional innovation. Segmentally duplicated pairs showed moderately elevated Ka/Ks ratios, with several approaching 0.4 (*GmLOX20-GmLOX38* = 0.398, *GmLOX17-GmLOX37* = 0.393), indicating relaxed constraints and potential subfunctionalization. The divergent stress-response patterns observed in our expression profiling support regulatory subfunctionalization as the primary diversification mechanism. Duplicated pairs with similar Ka/Ks ratios often possessed dramatically different promoter architectures (**Figure 10**), enabling functional partitioning through temporal, spatial, or conditional expression differences rather than catalytic changes. Seven *GmLOX* genes (*GmLOX5*, *GmLOX6*, *GmLOX15*, *GmLOX16*, *GmLOX43*, *GmLOX24*, *GmLOX28*) encode truncated proteins representing ongoing pseudogenization. The low pseudogenization rate (10%) compared to typical post-duplication gene loss (>50%) underscores strong selective pressure maintaining functional *GmLOX* repertoires. Temporal stratification in Ks values confirms a two-phase expansion model: tandem duplications (Ks: 0.673-1.079) occurred primarily after the recent WGD (~13 MYA), while segmental duplications (Ks: 1.095-1.568) originated during ancient polyploidy events. This dual-mechanism strategy generated complementary adaptive capacities: tandem duplications provide quantitative flexibility through dosage modulation, while segmental duplications contribute qualitative diversity through regulatory specialization. The exceptional 90% retention rate following WGD indicates oxylipin biosynthesis represents a trait under continuous positive selection, potentially reflecting multifaceted roles in stress responses, nitrogen fixation, and development. This “core-and-expansion” model exemplifies how polyploid crop. This pattern aligns with broader research indicating that plant gene family expansion involves multiple, complementary mechanisms across different evolutionary time scales (Flagel and Wendel, 2009).

### 4.3 Regulatory architecture enables functional specialization

The unprecedented regulatory complexity uncovered by our promoter analysis, revealing 47 distinct cis-regulatory element types across *GmLOX* genes, shows how gene family expansion can be combined with regulatory diversification to create functional specialization. The consistent presence of light-responsive elements (Box 4 in all genes) and the widespread occurrence of stress-responsive elements (ABRE in 94%, defense elements in 95%) indicate that environmental responsiveness is a core aspect of LOX gene evolution. This regulatory diversity differs from previous soybean *LOX* studies that mainly focused on seed-specific enzymes and their roles in flavor development (Gatehouse, 2002). While early research highlighted the importance of seed lipoxygenases *LOX1, LOX2*, and *LOX3* for oxidative stability and flavor (Wei et al., 2025), our analysis shows that most soybean *LOX* genes are involved in environmental stress responses rather than seed-specific functions. The discovery of genes with highly dense regulatory elements, such as *GmLOX39, GmLOX23*, and *GmLOX3*, each containing over 25 auxin-responsive elements, suggests the evolution of key hormone-responsive regulators within oxylipin biosynthesis networks. This combinatorial regulation enables precise control of oxylipin production to meet specific cellular needs and environmental challenges; a sophisticated regulation not observed in smaller gene families.

### 4.4 Functional specialization in environmental adaptation

Our expression profiling reveals advanced functional specialization in soybean *LOX* genes that goes beyond their traditional seed-focused roles. We identified five distinct stress response categories: alkaline-specific, drought-dominant, heat-specific, salt-responsive, and multi-stress responsive. This illustrates how gene family expansion can lead to the development of specialized adaptive abilities. The strongest responses occur under drought stress, with *GmLOX16* showing a 38-fold increase, likely due to water limitation being a key selective pressure in soybean evolution. This focus on drought tolerance aligns with agricultural needs for crops that can handle variable rainfall, supporting the idea that LOX gene expansion enhances environmental resilience. The timing of stress responses across three phases (early, intermediate, and late) suggests the development of integrated stress management systems. This advanced temporal regulation differs from simpler models where individual genes respond separately to stress signals. Instead, our data supports a model where the expanded *LOX* family enables coordinated, multi-phase responses that improve both immediate stress tolerance and long-term adaptation. Recent biotechnological techniques have started targeting specific LOX genes for crop improvement. For example, CRISPR/Cas9 targeting of the Lox-2 PLAT/LH2 domain can decrease seed lipoxygenase activity for better flavor (Patel et al., 2025). Our detailed functional analysis provides a basis for expanding such targeted strategies to boost both stress tolerance and crop quality.

### 4.5 Co-expression network reveals transcriptional regulation of *GmLOX* genes

The co-expression network analysis identified 64 TF-LOX interactions, revealing the transcriptional regulatory landscape of *GmLOX* genes. The presence of stress-responsive TF families (WRKY, NAC, AP2/ERF) co-expressed with *GmLOX* genes supports their role in stress adaptation. The co-expression with AP2/ERF TFs is particularly relevant given LOX enzymes’ role in jasmonate biosynthesis, suggesting feedback mechanisms coordinating oxylipin production with hormonal responses. The involvement of 77 different TFs regulating 43 *GmLOX* genes reflects the complexity arising from soybean genome duplications, likely contributing to functional specialization through regulatory divergence. Hub TFs co-expressed with multiple *GmLOX* genes, represent candidate master regulators warranting experimental validation. While co-expression suggests functional relatedness, direct regulatory interactions require validation through ChIP-seq or transactivation assays. Future studies should focus on experimental validation of key TF-LOX interactions; characterization of cis-regulatory elements in *GmLOX* promoters; and functional analysis through genetic manipulation. Understanding these regulatory networks has practical implications for soybean improvement, as manipulation of key TFs could enhance stress tolerance or modify seed quality traits.

### 4.6 Broader implications for crop genome evolution

The soybean LOX expansion exemplifies how polyploid crop genomes can maintain and exploit gene family expansions for enhanced environmental adaptation. Unlike many expanded families that undergo substantial gene loss through pseudogenization, the LOX family demonstrates how functional constraints and selective advantages can preserve large gene repertoires over evolutionary time (Iturbide et al., 2015). This pattern may be particularly relevant for understanding crop evolution in the context of climate change, where enhanced stress tolerance capabilities become increasingly valuable. The multiple duplication mechanisms, ancient whole-genome duplications followed by recent tandem duplications, illustrate the multi-layered nature of adaptive evolution in crop species, where different evolutionary processes contribute to adaptive potential at various timescales. The high level of functional retention within the expanded LOX family suggests that oxylipin-mediated signaling represents a core adaptive system that has been under continuous selective pressure throughout soybean evolution. This finding has implications for understanding which biological pathways are most likely to benefit from gene family expansion and which crops might harbor similar adaptive expansions.

## 5 Conclusions

The soybean LOX gene family exemplifies a unique case of adaptive gene family expansion, which has cultivated complex biological abilities that support crop adaptation to environmental changes. The combination of ancient whole-genome duplications, recent tandem expansions, regulatory diversification, and functional specialization has created an exceptionally versatile oxylipin biosynthetic system, far surpassing the complexity seen in other plant species. This case study demonstrates key principles of gene family evolution in polyploid crop genomes while offering practical tools for crop improvement. The retention of 43 functional LOX genes over evolutionary time highlights the vital role of oxylipin signaling in plant adaptation. It positions LOX genes as valuable targets for boosting agricultural resilience amid changing environmental conditions. Our comprehensive analysis lays a foundation for understanding the evolutionary forces shaping crop genomes and for developing biotechnological strategies for sustainable agricultural intensification.

## Data Availability

All sequence data used in this study are publicly available from the Phytozome database (https://phytozome-next.jgi.doe.gov/). The complete dataset, including gene sequences, annotation files, and analysis results are available as supplementary materials. Raw data and analysis scripts are available from the corresponding author upon reasonable request.
